# Army News

**Published:** 1918-12

**Authors:** 


**Army News.**

---

Dr. John S. Turner of Dallas, Texas, recently received a commission as captain in the Medical Reserve Corps with instructions to report to the commanding general at Camp Travis. On reporting for duty Captain Turner was assigned to the pyschiatric board.

Dr. J. K. Webster of Athens, has been commissioned first lieutenant in the Medical Corps and assigned to Camp Logan.

Dr. Harry A. McDaniel of Bonham, Texas, has been commissioned captain in the Medical Corps of the army, and has been ordered to report at the base hospital, Camp MacArthur, Waco.

Dr. John H. Brice, who has been in charge of the Lamesa Sanitarium for several years, has been appointed captain in the Medical Corps of the army and has gone to Fort Oglethorp, Camp Greenleaf, Ga., the medical officers' training camp.

Dr. Ross R. May of Whitewright, Texas, was recently commissioned a lieutenant in the United States Navy.

Dr. W. O. Stephenson of Dallas was lately commissioned captain in the Medical Corps and assigned to Camp John Wise, Air

Service, at San Antonio. Captain Stephenson is a Spanish-American War veteran; he served with the North Atlantic Squadron, which destroyed Cervera's fleet at Santiago, Cuba.

Lieutenant Henry Clay, well-known Dallas physician, has recently qualified as operating orthopedic surgeon. This qualification was made by his graduation from Harvard Graduate School of Medicine, Boston. He has been assigned to base hospital No. 159, France. He is awaiting orders to proceed overseas.

Dr. J. H. Stephenson of Dallas, recently volunteered his services, received a first lieutenant's commission, and was promptly assigned to Camp Logan, Houston.

Dr. John T. M. Finney of Baltimore, surgeon in chief of the American Expeditionary forces, who recently made a brief visit to this country, has returned to France after having secured a large appropriation from the Federal Government which places at his disposal several millions of dollars to erect and maintain a hospital in France for the treatment of shell-shocked American soldiers.

Captain Roy Saunders, who is stationed now at Camp Greenleaf, has been promoted recently to major, according to a message received by relatives in Fort Worth. Major Saunders is the son of Dr. and Mrs. Bacon Saunders.

Dr. J. V. Hopkins of Victoria, Texas, now a lieutenant in the Medical Corps of the United States Army, at Hattiesburg, Miss., returned to render professional service to those of his home town who were ill of influenza, his furlough having been granted upon request of the local Red Cross chapter.

Lieutenant G. Howard Spivey of Dallas, son of Dr. G. A. Spivey, visiting physician attached to the Emergency Hospital, was recently gassed while administering to wounded soldiers on the field of battle. Lieutenant Spivey is a member of the Medical Corps attached to the 315th Field Artillery. He enlisted in April, 1917, and went overseas in June of this year.

---

Have you received a bill for your subscription to the Texas Medical Journal within the last month? If so, what did you do with it? Perhaps you gave it the same attention some of your least appreciated patrons give to your bills. Treat others as you would have them treat you. If you have not paid your subscription, send it in at once, so that it will reach the editor before Christmas.

